# Association between bullying victimization and post-traumatic stress disorders among Chinese adolescents: a multiple mediation model

**DOI:** 10.1186/s12888-023-05212-x

**Published:** 2023-10-17

**Authors:** Tianchang Li, Bo Chen, Qian Li, Xinyue Wu, Yifan Li, Rui Zhen

**Affiliations:** 1 Jing Hengyi School of Education, Hangzhou Normal University, No. 2318 Yuhangtang Street, Hangzhou, 311121 China; 2 Faculty of Psychology, Beijing Normal University, Beijing, 100875 China; 3 Department of Psychology and Behavioral Sciences, Zhejiang University, Hangzhou, 310030 China; 4 Zhejiang Philosophy and Social Science Laboratory for Research in Early Development and Childcare, Hangzhou Normal University, Hangzhou, 311121 China

**Keywords:** Bullying victimization, Post-traumatic stress disorder, Social anxiety, Loneliness, Rumination

## Abstract

**Background:**

Research that focused on the mechanisms underlying the relation between school bullying victimization and PTSD ignored the simultaneous effect of emotional and cognitive factors, which may limit our comprehensive understanding of their roles. Besides, most researchers included non-bullying victims in data analysis, and this may mask the true effect among bullying victims. The present study aimed to explore the relation between bullying victimization and PTSD, and the mediating roles of social anxiety, loneliness, and rumination, after filtering out non-bullying victims.

**Materials and methods:**

In April 2019, we used convenience sampling to recruit 5013 students from Grade 10 and 11 in two high schools in Anhui Province, China. The mean age of these students was 16.77 (*SD* = 0.92) years. They completed five self-report questionnaires including the Delaware Bullying Victimization Scale-Student Chinese Revision (DBVS-S), the modified PTSD Checklist, the Social Anxiety Scale, the Adolescent Loneliness Scale, and the Rumination Scale. Further, a total of 443 bullying victims were screened out for this study according to the critical score of the DBVS-S.

**Results:**

The results showed that bullying victimization had a direct and positive association with PTSD among adolescents (*β* = 0.16, 95%CI: 0.046–0.252). Bullying victimization was positively associated with PTSD through increasing adolescents’ social anxiety (*β* = 0.06, 95%CI: 0.017–0.105), as well as through increasing their loneliness (*β* = 0.16, 95%CI: 0.109–0.215). In addition, bullying victimization was positively associated with PTSD through social anxiety via loneliness (*β* = 0.04, 95%CI: 0.013–0.067), as well as through loneliness via rumination (*β* = 0.02, 95%CI: 0.003–0.033). Bullying victimization was also positively associated with PTSD through a three-step path from social anxiety to rumination via loneliness (*β* = 0.004, 95%CI: 0.001–0.009).

**Conclusions:**

Social anxiety, loneliness, and rumination have important mediating effects in the relation between bullying victimization and adolescents’ PTSD, in which emotional factors (e.g., social anxiety, loneliness) are more crucial than cognitive factors (e.g., rumination). Intervention should pay more attention to timely alleviate victims’ emotional problems to reduce the risk of developing PTSD.

## Introduction

School bullying is a widespread and global problem. It is an intentional and repeated physical or psychological harm that occurs between individuals of imbalanced power [1]. School bullying includes verbal bullying (e.g., teasing), relational bullying (e.g., isolation), physical bullying (e.g., beatings), and cyberbullying (e.g., online harassment) [2]. Bullying victimization refers to the fact that students are repeatedly harmed by intentional aggressive behaviors of other powerful students [3]. The victims who are repeatedly bullied or abused over a long time tend to show severe physical or mental problems, including post-traumatic stress disorder (PTSD), depression [4], self-harm [5], and even suicide [6].

Post-Traumatic Stress Disorder (PTSD) is one of the most typical negative psychological outcomes after bullying victimization [7]. PTSD refers to a delayed and long-standing psychological or mental disorder caused by major psychological trauma events [8]. It involves four symptom clusters: intrusive symptom cluster (e.g., flashbacks), avoidance symptom cluster (e.g., active avoidance thoughts and situations), symptom cluster of negative cognition and emotion alteration (e.g., exaggerated negative thoughts about the world), and hyper-arousal symptom cluster (e.g., irritable behavior and angry outbursts) [9]. As a major traumatic event involving interpersonal violence [10, 11], school bullying may elicit PTSD symptoms among the victims [8, 12]. For example, Idsoe and colleagues found that 33.7% of bullying victims achieved clinically significant scores on the Trauma Symptom Scale. Hence, bullying victimization may be positively associated with PTSD symptoms [11].

In order to block or intervene in PTSD among adolescents, it is necessary to understand the internal mechanism underlying the relation between bullying victimization and PTSD. After encountering school bullying, the victims may experience emotional (e.g., social anxiety, loneliness) [13, 14] and cognitive problems (e.g., rumination) [15], which will further elicit PTSD symptoms [16, 17].

Social anxiety may play a mediating role in the relation between bullying victimization and PTSD. Social anxiety refers to irrational and excessive fear of interpersonal interactions and performance in social situations, and embodies intense social tension, distressed emotional experiences, and behavioral tendencies toward avoiding social interaction [18]. People with social anxiety may suffer from subjective experiences (e.g., pain, discomfort, fear and anxiety) and social avoidance behaviors in social situations due to the fear of negative evaluation [19]. School bullying victims are more likely to feel anxious in social situations [20], as they have developed negative self-image since being bullied is a very negative social experience [21]. In social situations, the victims recall their negative self-image [22] and believe that they will be negatively evaluated, thereby causing social anxiety [22]. Their social anxiety may further result in PTSD. Specifically, socially anxious individuals hold that expressing emotions is an act of weakness, so they choose not to express emotions [23] to avoid its negative consequences such as rejection, and negative evaluation [22]. Therefore, suffering from school bullying, the socially anxious victims may suppress their negative emotions, which is harmful to releasing emotions and coping with the traumatic event [24]. Eventually, related negative psychological reactions will be exacerbated, making it easier to develop PTSD symptoms [25].

In addition, loneliness may mediate the relation between bullying victimization and PTSD [13, 26]. Loneliness is a situation experienced by individuals when the number of existing relationships is fewer than what is considered desirable or admissible, as well as when the intimacy they desire has not been realized [27, 28]. The bullying victims suffered from a strong peer rejection, meaning their social needs are not fully met. Thus, victimization may lead to a stronger sense of loneliness [29]. Individuals with loneliness are more likely to be immersed in negative emotional states (e.g., pessimism, feelings of inferiority) [30] and negative cognition [31], hence lonely victims may develop a negative view of themselves, others, and the world, which may elicit PTSD symptoms [32].

Rumination may also be an important mediator in the relation between bullying victimization and PTSD. It is repetitive thinking about the causes and consequences of negative events or emotions [33]. These thoughts relate to the antecedents or nature of one’s negative affect, and are not goal directed and do not lead to plans for remedial action [34]. According to the shattered assumption theory [35], traumatic events (e.g., bullying victimization) may challenge individuals’ core beliefs system, cause cognitive imbalance, and induce repeated cognitive processing and repetitive thinking about the traumatic events and its negative outcomes, i.e., rumination [35]. Victims’ rumination may exacerbate the negative imaginings of the bullying event, which may further lead to PTSD symptoms [32, 36].

While social anxiety, loneliness, and rumination may mediate the relation between bullying victimization and PTSD, few studies to date have examined the combined associations among the three mediators. As a matter of fact, social anxiety may exacerbate rumination and loneliness [37]. According to the cognitive model of social phobia [38, 39], after social situations, individuals with social anxiety tend to do post-processing, such as reflecting on their shortcomings and mistakes [40]. Hence, they tend to ruminate when experiencing social stress [37, 40, 41]. Besides, socially anxious individuals are less accepted and supported by their peers [42], which leads them to have fewer close friends [42] and lower quality of relationship [43], thereby feeling lonely. Lonely individuals may also experience high levels of rumination [44]. As they have negative cognition about social interactions [30, 31, 45], they may experience more repeated stress and rumination [46].

In previous studies on the mechanism underlying the relation between bullying victimization and PTSD, emotional factors such as loneliness and social anxiety [47, 48] and cognitive factors such as rumination [49] have been evaluated separately. However, few simultaneously examined and compared the roles of emotional and cognitive factors, which may provide more targeted guidance for school bullying intervention. In addition, in many large-scale surveys, all the participants were included in data analysis even though most of them were not actually bullied. The results of non-bullied individuals may mask the true effect. Hence, we will screen participants and retain those who have been bullied as subjects to explore the influencing mechanism. More importantly, students from Grade 10 and 11 are around 16 and 17 years old, and school bullying occurs frequently at this age group [50]. Due to the high pressure of pursuing higher education, these students may be more susceptible to the negative impact of school bullying when being involved. Therefore, it is important for us to understand the mechanism by which bullying victimization develop into PTSD among students in this age group.

**Fig. 1:**
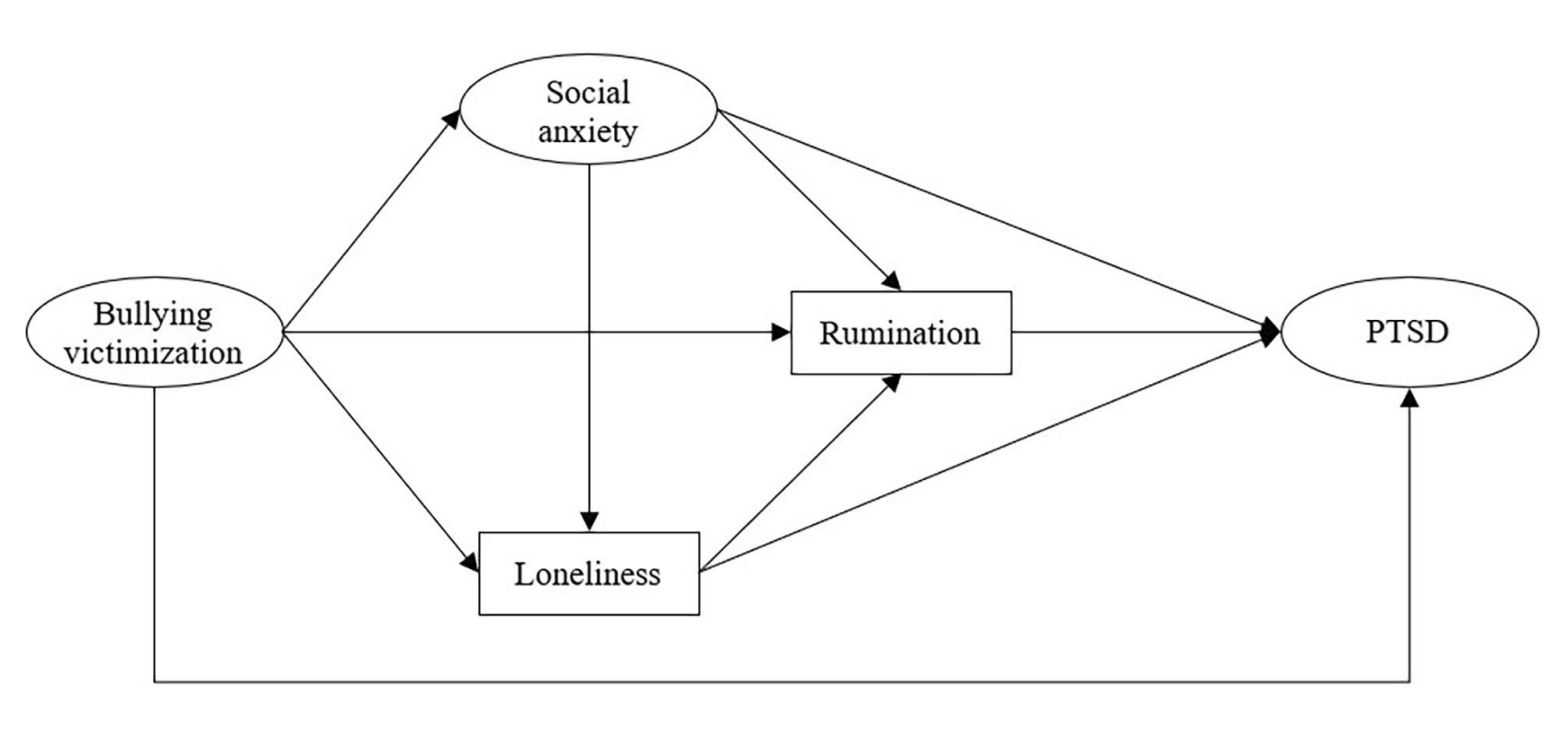
The hypothesized model of multiple mediating effects in the relation between bullying victimization and PTSD. PTSD = Post-traumatic stress disorder

Above all, we hypothesized that bullying victimization will be positively associated with PTSD through increasing social anxiety (H_1a_), loneliness (H_1b_), and rumination (H_1c_), respectively. Bullying victimization will be positively associated with PTSD through the two-step paths from social anxiety to loneliness (H_2a_), from social anxiety to rumination (H_2b_), and from loneliness to rumination (H_2c_), respectively. Bullying victimization will be positively associated with PTSD through a three-step path from social anxiety to rumination via loneliness (H_3_). The hypothesized model of mediating effects is shown in Fig. 1.

## Methods

### Participants and procedures

This study used convenience sampling to recruit participants. In April 2019, after obtaining the approval of school principals of two senior high schools in Anhui Province, China, we recruited all students from Grade 10 and 11 who attended school on the investigation day. Students from Grade 12 were not recruited because of their busy academic schedule. In total, 5013 students participated in our paper-and-pencil investigation, of which 2410 were boys, 2450 were girls, and 153 did not report their sex; 2531 were from Grade 10, 2480 were from Grade 11, and 2 did not report their grade; the mean age of participants was 16.77 years (*SD* = 0.92 years).

When students scored 2 or more on any item of the Delaware Bullying Victimization Scale-Student Chinese Revision, they were considered to have been bullied [51]. Based on this, 443 (8.8%) participants met the bullying victimization criteria. We used the data of the 443 bullying victims for further analysis and discussion. Demographic information is shown in Table 1.

This study was approved by the Research Ethics Committee of the School of Medicine, Zhejiang University (No. 2019-051). Students’ participation is voluntary and anonymous, and they can choose to withdraw from the survey at any time. Informed consent was obtained from their guardians. All procedures performed in this study involving human participants were in accordance with the ethical standards of the research committee. We conducted this investigation in a classroom setting. Participants had about 30 min to finish the questionnaires. No compensation was provided for any participants.

**Table 1:** Demographic information of 443 bullying victims

Variable	Number (proportion)
Age	*M* ± *SD*: 16.77 ± 0.92
Gender	
Male Female Unreported	322 (72.69%) 106 (23.93%) 15 (3.38%)
Grade	
10 11 Unreported	228 (51.47%) 214 (48.31%) 1 (0.22%)
Parents’ marital status	
Normal Divorced Widowed Unreported	390 (88.04%) 28 (6.32%) 7 (1.58%) 18 (4.06%)
Parents’ working situation	
Both parents work in other cities Father works in other cities Mother works in other cities Both parents work locally Unreported	107 (24.15%) 93 (20.99%) 5 (1.13%) 216 (48.76%) 22 (4.97%)
Main caregivers	
Father Mother Grandparents Other relatives Unreported	66 (14.90%) 211 (47.63%) 107 (24.16%) 39 (8.80%) 20 (4.51%)

### Measures

***Bullying victimization.*** Bullying victimization was measured with the Delaware Bullying Victimization Scale-Student Chinese Revision (DBVS-S) [52]. Bear and colleagues [53] developed the original questionnaire, and Xie and colleagues [52] revised it and developed the Chinese version. We used the Chinese version to ensure the instrument was suitable for Chinese students. The scale has a four-factor structure with 17 items including verbal bullying, physical bullying, relational bullying, and cyberbullying. The 13th item “I was bullied at this school” was not included in data analysis [54]. The scale is scored by a 6-point Likert scale from 0 (never) to 5 (every day). When students scored 2 or more on any item of the scale, they were considered to have been bullied [51]. In this study, the internal reliability of the scale was good (Cronbach’s α = 0.91).

***Social anxiety.*** Social anxiety was measured with the Social Anxiety Scale developed by La Greca and colleagues [55]. The scale has 10 items with a two-factor structure including fear of negative evaluation, and social avoidance and distress. The scale is scored by a 4-point Likert scale from 0 (never) to 3 (always). A higher score indicates a higher level of social anxiety. In this study, the internal reliability of the scale was good (Cronbach’s α = 0.87).

***Loneliness.*** Loneliness was measured with the Adolescent Loneliness Scale Chinese Revision [56]. Asher and colleagues [57] developed the original questionnaire, and Zou and colleagues [56] revised it and developed the Chinese version. The scale has 21 items with a four-factor structure including feelings of loneliness, subjective estimations of peer status, feelings of social adequacy versus inadequacy, and perceived social competence. We used the feelings of loneliness subscale in this study. The scale is scored by a 5-point Likert scale from 1 (not true at all) to 5 (always true). A higher score indicates a higher level of feelings of loneliness. In this study, the internal reliability of the feelings of loneliness subscale was good (Cronbach’s α = 0.86).

***Rumination.*** Rumination was measured with the rumination subscale of the Chinese version of the Cognitive Emotion Regulation Questionnaire (CERQ-C) [58]. Garnefski and colleagues [59] developed the original questionnaire, and Zhu and colleagues [58] translated it and developed the Chinese version. The rumination subscale contains 3 items, which were scored by a 5-point Likert scale from 0 (Never) to 4 (Always). A higher score indicates a higher level of rumination. In this study, the internal reliability of the rumination subscale was acceptable (Cronbach’s α = 0.73).

***Post-traumatic Stress Disorder.*** PTSD was measured with the modified PTSD Checklist for DSM-5 translated and revised by Zhou and colleagues [60]. The scale has 20 items with a four-factor structure including intrusive symptoms, avoidance symptoms, negative alterations of cognition and moods, and hyperarousal symptoms. The scale is scored by a 5-point Likert scale from 0 (completely inconsistent) to 4 (completely consistent), with a total score of 0 ~ 80 and 31 as the demarcation criteria for PTSD symptoms. A higher score indicates a higher level of PTSD. In this study, the internal reliability of the scale was good (Cronbach’s α = 0.90).

### Data analysis strategies

We used SPSS 25.0 to calculate the means, standard deviations, Pearson’s correlation coefficients, and the internal reliability coefficients of scales. Little’s Missing Completely at Random (MCAR) test revealed the data were missing at random [*χ*² (37) = 22.54, *p* = 0.97]. The normality test showed that only PTSD was normally distributed (*p* = 0.15), while other measures, including bullying victimization, social anxiety, loneliness, and rumination, were not normally distributed. The skewness and kurtosis coefficients of bullying victimization were greater than 2 and 7, respectively, while those of other measures were all less than 2. Therefore, missing data were handled with maximum likelihood robust estimates (MLR) when building models. We firstly assessed the measurement model. Further, we performed structural equation modeling analysis using Mplus 8.0 to examine the mediating role of social anxiety, loneliness, and rumination. Specifically, we conducted a two-step procedure to examine the chain mediating roles of social anxiety, loneliness, and rumination in the relation between bullying victimization and PTSD. First, we developed a direct effect model to assess the association between bullying victimization and PTSD. Second, based on the direct effect model, we inserted social anxiety, loneliness, and rumination as mediators into the relation between bullying victimization and PTSD to form a chain mediating effects model. The chain mediating effects model was evaluated by the model fit indices (e.g., *χ*^2^, CFI, TLI, RMSEA, and SRMR). CFI and TLI values larger than 0.90, and RMSEA and SRMR values less than 0.08 indicate an acceptable model fit.

## Results

### Descriptive statistics and correlations among main measures

Descriptive characteristics and correlations among all measures are shown in Table 2. The mean levels of bullying victimization, social anxiety, loneliness, rumination, and PTSD were 10.86 (0 ~ 80), 10.35 (0 ~ 20), 15.73 (6 ~ 30), 6.88 (0 ~ 12), and 39.74 (0 ~ 80), respectively. In addition, we found that gender was positively associated with social anxiety, loneliness and PTSD, but age had no significant relation with other variables. The correlation between bullying victimization and rumination was marginally significant (*p* = 0.065), and correlations between all other main variables were significant, indicating bullying victimization was significantly and positively associated with PTSD, social anxiety, loneliness, and rumination.

**Table 2:** Means, standard deviations, and correlations among main variables

Variables	*M* (*SD*)	1	2	3	4	5	6	7
1.Gender		1						
2.Age	16.77 (0.92)	-0.01	1					
3.Bullying victimization	10.86 (11.54)	-0.08	0.02	1				
4.Social anxiety	10.35 (4.74)	0.15^**^	0.00	0.18^***^	1			
5.Loneliness	15.73 (5.65)	0.11^*^	0.08	0.39^***^	0.42^***^	1		
6.Rumination	6.88 (2.64)	-0.01	0.08	0.09^#^	0.20^***^	0.26^***^	1	
7.PTSD	39.74 (14.21)	0.12^*^	0.09	0.33^***^	0.42^***^	0.62^***^	0.38^***^	1

Note.^*^p < 0.05, ^**^p < 0.01, ^***^p < 0.001, ^#^*p* = 0.065, PTSD = Posttraumatic Stress Disorder

### Analysis of measurement model

**Fig. 2:**
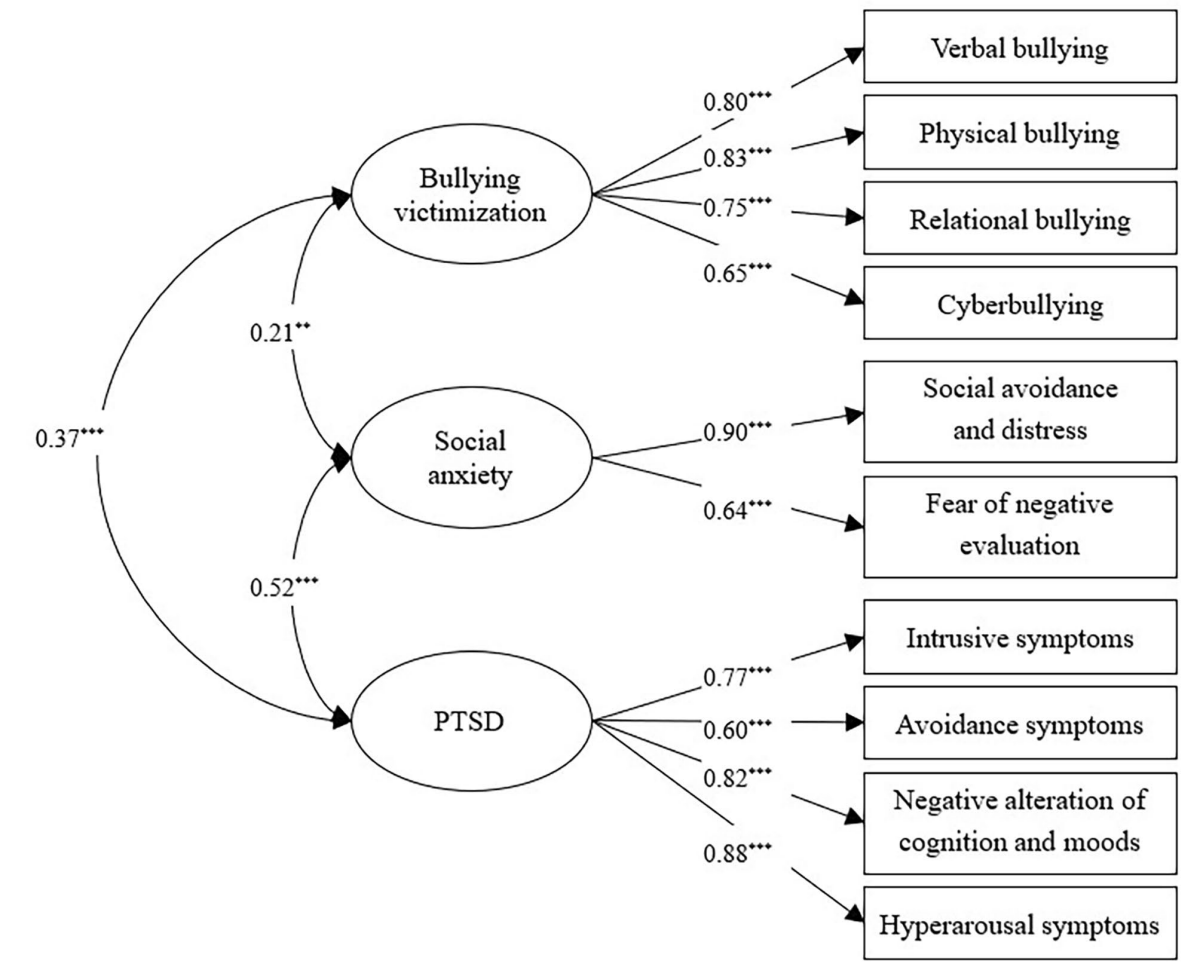
Measurement model

We used Confirmatory Factor Analysis to test the measurement model, and the measurement model was acceptable [*χ*^2^(32) = 108.921, CFI = 0.947, TLI = 0.925, RMSEA (90% CI) = 0.074 (0.059–0.089), SRMR = 0.041] (see Fig. 2).

### Analysis of Direct Effect Model

Prior to the analysis of the indirect effects model, we first examined the direct role of bullying victimization on PTSD. The direct effect model was acceptable [*χ*^2^ (25) = 98.645, CFI = 0.938, TLI = 0.911, RMSEA (90%CI) = 0.083 (0.066–0.101), SRMR = 0.043]. The results revealed that bullying victimization had a positive and significant effect on PTSD (*β* = 0.43, *p* < 0.001).

### Analysis of multiple Mediating Effects Model

**Fig. 3:**
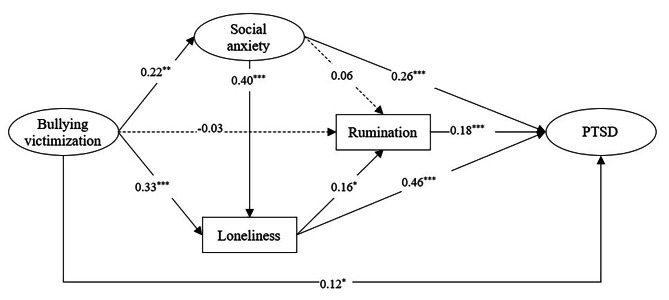
The model of multiple mediating effects in the relation between bullying victimization and PTSD. ^*^p < 0.05, ^**^p < 0.01, ^***^p < 0.001

Based on the direct effect model, we added social anxiety, loneliness, and rumination as mediators between bullying victimization and PTSD after controlling for gender and age (see Fig. 3). The multiple indirect effects model was also acceptable [*χ*^2^(46) = 153.927, CFI = 0.937, TLI = 0.910, RMSEA (90% CI) = 0.073 (0.060–0.086), SRMR = 0.040]. The results showed that bullying victimization was positively associated with PTSD (*β* = 0.12, *p* < 0.05). For mediating effects, bullying victimization was indirectly associated with PTSD through social anxiety and loneliness as mediators, respectively, but not through rumination. Bullying victimization was associated with PTSD through the two-step paths from social anxiety to loneliness and from loneliness to rumination. Bullying victimization was associated with PTSD through a three-step path from social anxiety to rumination via loneliness.

Furthermore, we conducted bias-corrected bootstrap tests with a 95% confidence interval to evaluate the significance of the indirect effects in Fig. 3, and the results were shown in Table 3. A 95% confidence interval of an indirect path coefficient that does not include 0 is suggested to be significant. For example, as shown in Table 3, the 95% confidence interval of the indirect path from bullying victimization to PTSD via loneliness (0.109, 0.215) did not include 0, indicating that the indirect path was significant. The 95% confidence interval of the indirect path from bullying victimization to PTSD via rumination (-0.037, 0.027) includes 0, indicating that the indirect path was not significant.

**Table 3:** Bias-corrected bootstrap test on mediating effects

Paths	*β*	95% CI	
		Low	High
Bullying victimization→PTSD	0.16^**^	0.046	0.252
Bullying victimization→Loneliness→PTSD	0.16^***^	0.109	0.215
Bullying victimization→Rumination→PTSD	-0.004	-0.037	0.027
Bullying victimization→Social anxiety→PTSD	0.06^*^	0.017	0.105
Bullying victimization→Social anxiety→Loneliness→PTSD	0.04^**^	0.013	0.067
Bullying victimization→Loneliness→Rumination→PTSD	0.02^*^	0.003	0.033
Bullying victimization→Social anxiety→Rumination→PTSD	0.01	-0.001	0.015
Bullying victimization→Social anxiety→Loneliness→Rumination→PTSD	0.004^#^	0.001	0.009

Note.^*^p < 0.05, ^**^p < 0.01, ^***^p < 0.001, PTSD = Posttraumatic Stress Disorder

## Discussion

We screened bullying victims out from a large sample, and examined the mechanism underlying how bullying victimization was associated with PTSD through social anxiety, loneliness, and rumination as mediators. The results showed that bullying victimization was significantly and positively associated with PTSD. This is consistent with previous studies [11, 61] and suggests that school bullying should be considered as a traumatic event. Based on the cognitive evaluation theory [62], school bullying is a serious traumatic event for the victims [16], and it is difficult for them to positively evaluate this traumatic event in a short period of time [62]. As a result, the victims may be fearful of the scenes of being bullied before, nervous and helpless that they will be harmed again in the future, and gradually develop PTSD symptoms [63].

The results also showed that social anxiety played a mediating role between bullying victimization and PTSD, which supported H_1a_. According to the IAM model (Integrated Aetiological and Maintenance Model of Social Anxiety Disorder) [64], negative experiences with peers (e.g., bullying victimization) may increase students’ level of threat assessment in social situations. In other words, victims are more likely to perceive social situations as threatening, to think that others will negatively evaluate them, which leads to social anxiety [64]. People with social anxiety have a negative biased interpretation of events. Specifically, they interpret positive, neutral, and ambiguous events as negative, and interpret negative events as catastrophic [22]. Hence, adolescents with social anxiety may negatively and catastrophically evaluate traumatic events, which will result in PTSD symptoms [32].

Loneliness also played a mediating role between bullying victimization and PTSD, which supported H_1b_. Bullying victims experienced abusive interpersonal interactions. As a result, they may feel fearful, insecure, and distrustful of social partners [65], which is detrimental to the development of peer relationship [66], thereby feeling lonely. Lonely adolescents feel threatened in their daily life, and they are wary of social interactions and stay away from social partners [30, 31]. They have nobody to confide in about their experience of being bullied, to pour out negative emotions, and to gain understanding and support. These all may lead to PTSD symptoms [24].

Nevertheless, the mediating effect of bullying victimization on PTSD through rumination was not significant due to the non-significant path from bullying victimization to rumination. H_1c_ was not supported and the result was inconsistent with the shattered assumption theory [67]. It is noteworthy that the victims did not show rumination directly after bullying victimization, but developed rumination after they experienced loneliness. Specifically, bullying victims may feel unacceptable, excluded and incompetent, resulting in negative self-evaluation and feelings of inferiority [68]. Therefore, in order to hide their deficiencies, victims may limit the disclosure of personal information, feelings and ideas, and thus isolate themselves from social relationships [69] and become lonely. People with high levels of loneliness are more likely to adopt negative coping styles and response styles [44]. They attribute interpersonal failure to themselves and believe that they lack the ability to change the situation [70]. Therefore, instead of solving problems [71], they may indulge in negative emotions and constantly think about the causes, meanings and consequences of negative emotions, that is, rumination. Based on the above, we believe that the loneliness of bullying victims deserves attention.

In addition, bullying victimization was associated with PTSD through a two-step path from social anxiety to loneliness, which supported H_2a_. Bullying victimization increases the risk of social anxiety. According to the social selection model [72], people with social anxiety are less likely to attract and/or maintain positive interpersonal relationships than people without social anxiety [72]. Social anxiety hinders the establishment and maintenance of interpersonal relationships and reduces the quantity and quality of interpersonal relationships, leading to feelings of loneliness, and further eliciting PTSD symptoms.

Notably, the indirect effect of bullying victimization on PTSD through the two-step path from social anxiety to rumination was not significant, because the path from social anxiety to rumination was not significant. This result did not support H_2b_ and was inconsistent with the cognitive model of social anxiety [38, 39]. On the one hand, adolescents with social anxiety tend to reflect on their deficiencies, mistakes, and imperfections in social performance after social experiences, thus exhibiting ruminative thinking [40]. On the other hand, adolescents with social anxiety may also engage in anticipatory processing before social experiences. For instance, they may plan what to say, rehearse what to say, and even imagine what may happen and how to handle it [22]. These may help them to perform well in social situations [73] and subsequently reflect less on their performance. Above all, the effect of social anxiety on rumination was not significant. In addition, the mediating role of loneliness in the relation between social anxiety and rumination might make the above path insignificant. We found in this study that bullying victimization was associated with PTSD through a three-step path from social anxiety to rumination via loneliness. That is, social anxiety did not directly predict rumination, but social anxiety was associated with rumination through increasing loneliness.

Additionally, bullying victimization was associated with PTSD through a two-step path from loneliness to rumination. This result supported H_2c_ and was consistent with previous studies [44]. Lonely adolescents are more likely to hold negative cognitions [30, 31, 45], which easily triggers rumination [46] and hence leads to PTSD. This result explained to some extent how loneliness increased PTSD via rumination. As mentioned above, bullying victimization was associated with PTSD through a three-step path from social anxiety to rumination via loneliness, which supported H_3_. This means that whether bullying victims develop PTSD or not largely depends on the victims’ cognition and emotional responses to the traumatic event [16, 63].

Some limitations of this study need to be addressed. First, all variables were based on self-report scales, so the results may be affected by common methodological biases. Diverse methods (e.g., experiments, interviews) are encouraged in the future. Second, we only screened participants who have been victimized, and neglected those who were both bullying victims and perpetrators. Third, the role of rumination in this study was different from previous findings related to traumatic events [74], possibly because school bullying is a special kind of traumatic event that involves peer violence and repeated harm. Researchers can further examine the inconsistent results. Finally, the findings did not reveal causal relations among variables due to the cross-sectional design.

Despite these limitations, we screened 443 bullying victims out of 5013 adolescents and examined the mechanism underlying how bullying victimization was associated with PTSD via the joint mediating roles of emotional and cognitive factors. The study found that social anxiety and loneliness played more important mediating effect than rumination did. That is, alleviating social anxiety and loneliness is more effective in preventing bullying victims from suffering from PTSD. The findings suggest targeted interventions. In specific, schools can establish an “Anti-bullying Union” formed by teachers and students, to monitor potential bullying behaviors and provide timely support for bullying victims. Psychological teachers can carry out individual counseling and students can provide companionship in time for victims, to relieve social anxiety and loneliness, thereby reducing their rumination, to reduce the risk of developing PTSD.

## Conclusion

This study has investigated the underlying mechanism how bullying victimization was related to PTSD in a sample of school bullying victims. The results indicated that emotional factors (e.g., social anxiety, loneliness) and cognitive factors (e.g., rumination) both served as mediators in this relation. However, rumination did not play a mediating role in this relation unless the emotional factors (e.g., social anxiety, loneliness) worked together with it. Therefore, intervention should prioritize focusing on emotional issues such as social anxiety and loneliness in bullied adolescents to alleviate subsequent adverse psychological outcomes like rumination and PTSD.

## Data Availability

The datasets used during the current study available from the first author on reasonable request.

## Declarations

All the research procedures involving human participants followed the Declaration of Helsinki in 1964 and its subsequent amendments. Informed consent was obtained from all the participants in the study. Informed consent was also obtained from the parents or legal guardian(s) of the participants under 16 years of age.
